# Phosphoproteomic Analysis of Thermomorphogenic Responses in *Arabidopsis*

**DOI:** 10.3389/fpls.2021.753148

**Published:** 2021-09-16

**Authors:** Yu-Jian Shao, Qiao-Yun Zhu, Zi-Wei Yao, Jian-Xiang Liu

**Affiliations:** State Key Laboratory of Plant Physiology and Biochemistry, College of Life Sciences, Zhejiang University, Hangzhou, China

**Keywords:** *Arabidopsis*, hypocotyl growth, warm temperature, phosphoproteomics, thermomorphogenesis

## Abstract

Plants rapidly adapt to elevated ambient temperature by adjusting their growth and developmental programs. To date, a number of experiments have been carried out to understand how plants sense and respond to warm temperatures. However, how warm temperature signals are relayed from thermosensors to transcriptional regulators is largely unknown. To identify new early regulators of plant thermo-responsiveness, we performed phosphoproteomic analysis using TMT (Tandem Mass Tags) labeling and phosphopeptide enrichment with *Arabidopsis* etiolated seedlings treated with or without 3h of warm temperatures (29°C). In total, we identified 13,160 phosphopeptides in 5,125 proteins with 10,700 quantifiable phosphorylation sites. Among them, 200 sites (180 proteins) were upregulated, while 120 sites (87 proteins) were downregulated by elevated temperature. GO (Gene Ontology) analysis indicated that phosphorelay-related molecular function was enriched among the differentially phosphorylated proteins. We selected ATL6 (ARABIDOPSIS TOXICOS EN LEVADURA 6) from them and expressed its native and phosphorylation-site mutated (S343A S357A) forms in *Arabidopsis* and found that the mutated form of ATL6 was less stable than that of the native form both *in vivo* and in cell-free degradation assays. Taken together, our data revealed extensive protein phosphorylation during thermo-responsiveness, providing new candidate proteins/genes for studying plant thermomorphogenesis in the future.

## Introduction

Plants are sessile organisms and able to adapt to changing environments, including diurnal and seasonal temperature fluctuations. In the model plant *Arabidopsis*, warm temperatures promote hypocotyl/petiole elongation and/or accelerate flowering, in a process called thermomorphogenesis (Casal and Balasubramanian, 2019). Several thermosensors were reported (Vu et al., 2019a), and they perceive warm temperature cues, have property changes at molecular level, and convey the temperature information to downstream components, for example, the key transcription factor phytochrome interacting factor 4 (PIF4; Vu et al., 2019b). Phytochromes were initially identified as photo-interconvertible photoreceptors that undergo conformation changes from the inactivated Pr form to the activated Pfr form upon absorbing red light, and dark exposure can revert this process (Pham et al., 2018). Recent studies have shown that phytochrome B (phyB) is also an important thermosensor, and warm temperature accelerates the conversion of phyB from an active Pfr form to an inactive Pr form, lifting the inhibitory effects of phyB on PIF4 (Jung et al., 2016; Legris et al., 2016). RNA structures are dynamic and often subjected to environmental temperature perturbation. Surprisingly, the secondary structure of *PIF7* RNA adopts a more relaxed, yet distinct conformation under warmer temperature conditions, leading to an enhanced protein translation of PIF7 in *Arabidopsis* (Chung et al., 2020). PIF7 acts as a transcription factor and plays a similar role to PIF4, inducing the expression of downstream genes involved in auxin biosynthesis and signaling (Chung et al., 2020; Fiorucci et al., 2020). EARLY FLOWERING3 (ELF3) is a required component of the core circadian clock (Thines and Harmon, 2010). It also plays important roles in warm temperature-induced hypocotyl elongation in *Arabidopsis* by inhibiting the expression of *PIF4* as well as inhibiting the protein activity of PIF4 (Nomoto et al., 2012; Box et al., 2015; Nieto et al., 2015; Raschke et al., 2015). Recently, ELF3 was proposed to function as a thermosensor (Jung et al., 2020). The polyQ tract of ELF3 resembles a prion-like domain and has a liquid-liquid phase separation (LLPS) change in response to increasing temperature, releasing the inhibitory effects of ELF3 on PIF4 (Jung et al., 2020). The ELF3 protein accumulation is attenuated by warm temperatures, which is controlled by XBAT31/35-mediated ubiquitination and protein degradation (Ding et al., 2018; Zhang et al., 2021a,c).

The bHLH transcription factor PIF4 is a central regulator for thermomorphogenic changes in plants by promoting the auxin biosynthesis and signaling (Koini et al., 2009; Franklin et al., 2011; Kumar et al., 2012; Sun et al., 2012; Proveniers and van Zanten, 2013). It is subjected to various regulations at both transcriptional level and post-translational level (Vu et al., 2019b). BRASSINAZOLE-RESISTANT 1 (BZR1), a key transcription factor in brassinosteroid (BR) signaling, also promotes the expression of *PIF4* and other temperature responsive genes under elevated ambient temperature (Oh et al., 2012; Ibanez et al., 2018). BRI1 EMS SUPPRESSOR 1 (BES1), another important BR responsive transcription factor, interacts with PIF4 and activates the expression of BR biosynthetic genes to promote hypocotyl growth under warm temperature conditions (Martinez et al., 2018). ELONGATED HYPOCOTYL5 (HY5) is a bZIP transcription factor that inhibits hypocotyl elongation by competing with PIF4 to bind to downstream targets (Gangappa and Kumar, 2017). In response to warm temperatures, HY5 is rapidly degraded by the ubiquitin E3 ligase COP1 because of more accumulation of COP1 in the nucleus under such conditions, which minimizes the inhibitory effect of HY5 on hypocotyl growth (Osterlund et al., 2000; Delker et al., 2014; Gangappa and Kumar, 2017; Park et al., 2017).

Molecular mechanisms underlying plant thermomorphogenesis are emerging (Zhang et al., 2021b); however, previous researches are more focused on transcriptional control in thermomorphogenesis, and the cellular signaling cascades downstream of the thermosensors are still largely unknown. Quantitative phosphoproteomics has been proved to be a powerful and versatile platform to identify new signaling components in a large scale, and new phosphorylation sites of proteins could be critical for their biological functions (Wang et al., 2013). To date, quantitative phosphoproteomics mainly relies on two major techniques, label-free quantitation and stable isotope labeling (Paulo and Schweppe, 2021). Recently, TMT (Tandem Mass Tags)-based labeling proteomics has provided a powerful quantitative platform with a high-sensitivity and accuracy, which has been widely used for studying proteome changes during plant growth, development, and responses to environmental conditions (Zhao et al., 2019; Yang et al., 2020; Chen et al., 2021). In this study, we performed the TMT-based quantitative phosphoproteomic analysis of plant thermo-responsiveness and provided information on temperature-responsive phosphorylation changes in plants. We believed that the data presented here not only revealed potential new regulators in thermomorphogenesis but also provided a large number of phosphorylation sites that are potentially critical for their function in thermo-responsiveness in plants.

## Materials and Methods

### Plant Materials and Hypocotyl Length Measurements

All *Arabidopsis* plants in the current study were in Columbia-0 (Col-0) background. Seeds were surface-sterilized with NaClO for 15min and washed three times with sterilized water. All the seeds were stratified at 4°C for 4days, after which they were transferred to half-strength Murashige and Skoog (MS) medium (containing 1.2% sucrose and 0.6% agar, pH 5.7) and grown in plant incubator in the dark. For phenotypic assays, seedlings were grown at 22°C for 3days, after which they were transferred to 29°C or maintained at 22°C for 4days. Representative plants were photographed, and hypocotyl length was measured with the aid of Image J. One-way analysis of variance (ANOVA) analyses and Tukey’s *post hoc* test (*p*<0.05) were done with the software Statistical Product and Service Solutions (SPSS).

### Quantitative Proteomics and Phosphoproteomics

Stratified wild-type (WT) seeds were exposed to light for 8h and then were kept in the dark at 22°C for 2days, after which the plates were kept at 22°C or 29°C for 3h, and the etiolated seedlings were sampled for further study with three biological replicates. Proteomics analysis was carried out in the company PTM BIO (Hangzhou, China). Briefly, samples were ground with the lysis buffer (8M urea, 1% Triton-100, 10mM dithiothreitol, 1% Protease Inhibitor Cocktail, and 1% phosphatase inhibitor), followed by sonication three times on ice using a high intensity ultrasonic processor (Scientz, Ningbo, China). The remaining debris was removed by centrifugation at 5,500*g* at 4°C for 10min. Finally, the protein was precipitated with cold 0.1M ammonium acetate overnight. After centrifugation at 12,000*g* 4°C for 10min, the supernatant was discarded. The remaining precipitate was washed with cold acetone for three times. The protein was redissolved in 8M urea, and the protein concentration was determined with BCA kit according to the manufacturer’s instructions. After digestion with trypsin, peptides were desalted by Strata X C18 SPE columns (Phenomenex, CA, United States) and labeled with TMT kits according to the manufacturer’s protocol. For phosphoproteomic study, phosphopeptides were enriched with TiO_2_ (IMAC). The tryptic peptides were dissolved in 0.1% formic acid and 2% acetonitrile (solvent A) and directly loaded onto a home-made reversed-phase analytical column (15-cm length, 75μm i.d.). The gradient increased from 4 to 22% solvent B (0.1% formic acid in 90% acetonitrile) over 38min, 22 to 32% in 14min, climbing to 80% in 4min, and then holding at 80% for the last 3min, all at a constant flow rate of 350nl/min on an EASY-nLC 1200 UPLC system. The peptides were subjected to NSI source followed by tandem mass spectrometry (MS/MS) in Q ExactiveTM Plus (ThermoFisher, MA, United States) coupled online to the UPLC system. The electrospray voltage applied was 2.2kV. The *m*/*z* scan range was 350–1,600 for full scan, and intact peptides were detected in the Orbitrap at a resolution of 60,000. Peptides were then selected for MS/MS using NCE setting as 28 and the fragments were detected in the Orbitrap at a resolution of 17,500. A data-dependent procedure that alternated between one MS scan followed by 20 MS/MS scans with 15.0s dynamic exclusion. Automatic gain control (AGC) was set at 1E5. Fixed first mass was set as 100*m*/*z*. The resulting MS/MS data were processed using the Maxquant search engine (v.1.5.2.8) against the *Arabidopsis* TAIR database. The mass error for precursor ions was less than 20ppm in the first search and 5ppm in the main search, and the mass error for fragment ions was less than 0.02Da. Differentially regulated phosphopeptides were calculated and their corresponding proteins were subjected to GO analysis (Biological Process, Cellular Component, Molecular Function).

### Plasmid Construction and Mutagenesis

For overexpression of *ATL6*, the coding sequence of *ATL6* was amplified and inserted into pCambia1306 with the 35S CaMV promoter, and the FLAG tag was fused to ATL6 at the N-terminus. The mutated form of ATL6 was created by overlapping PCR. Error free plasmids were transformed into *Agrobacterium tumefaciens* strain GV3101 *via* the freeze-thaw method and introduced into *Arabidopsis* plants *via* the floral-dip method (Clough and Bent, 1998). All the primers used are listed in Supplementary Table S1.

### Cell-Free Degradation Assay and Western Blot Analysis

For cell-free degradation assay, the native and mutated forms of *ATL6* overexpression plants grown at 22°C in the dark for 3days were harvested and total proteins were extracted using the extraction buffer (20mM Tris-HCl, pH 7.4, 25mM NaCl, and 0.01% Nonidet P-40). After that the protein mixtures were incubated with 2mM ATP, 10μg/μl ubiquitin, and 50μM cycloheximide (CHX) at 30°C for 0–120min in the presence or absence of 200μM MG132. The reaction was stopped with 5× SDS buffer. For protein stability assay, total proteins were extracted with the extracting buffer [125mM Tris-HCl (pH 8.0), 375mM NaCl, 2.5mM EDTA, 1% SDS, 1% β-mercaptoethanol, and protease inhibitor cocktail complete tablets]. Afterward, the proteins were separated in 4–20% SDS-PAGE gels and analyzed using *anti*-FLAG (Abmart, Shanghai, China) and *anti*-tubulin (Sigma, CA, United States), respectively.

### RT-PCR

For RT-PCR, total RNA of the whole seedlings was extracted using an RNA Prep Pure Plant kit (Tiangen, Beijing, China) and reverse transcribed using M-MLV reverse transcriptase (Invitrogen, Shanghai, China) with oligo (dT) primers. RT-PCR was performed in a C1000 Touch thermal cycler (Bio-Rad, CA, United States). All the primers used are listed in Supplementary Table S1.

## Results

### Thermo-Responsive Hypocotyl Growth of *Arabidopsis* Plants in the Dark

**Figure 1:**
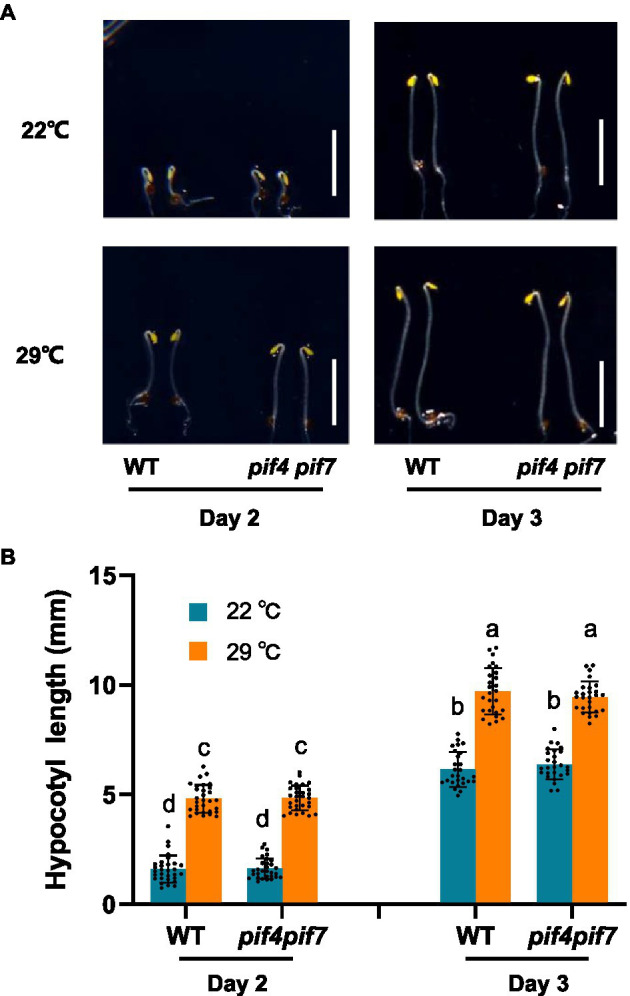
Thermo-responsive hypocotyl growth of *Arabidopsis* wild-type and *pif4 pif7* mutant plants. Wild-type (WT) and *pif4 pif7* double mutant plants were grown at 22°C or 29°C in darkness for the indicated time and photographed **(A)**, the hypocotyl length of each plant was subsequently measured **(B)**. The bars depict the SD (*n*=27). Letters above the bars indicate significant differences as determined by HSD test (*p*<0.05). Bar=5mm.

To prevent possible interference of photosynthesis-related proteins to our proteomic analysis of thermo-responsiveness, we firstly checked whether *Arabidopsis* plants could respond to ambient elevated temperatures in the dark in our experimental conditions. We grew *Arabidopsis* WT plants both at 22 and 29°C and monitored their hypocotyl length. Indeed, WT plants were responsive to ambient elevated temperature in terms of hypocotyl growth (Supplementary Figure S1). Previous results have shown that *pif4* and *pif7* loss-of-function mutants were unresponsive to increased ambient temperatures under light conditions (Fiorucci et al., 2020). In order to know whether these two transcription factors are also involved in thermo-responsiveness in the dark, we compared thermo-responsive hypocotyl growth between WT and *pif4 pif7* double mutant plants. It was found that both WT and *pif4 pif7* double mutant plants responded to elevated temperatures with a similar extent in the dark (Figures 1A,B). These results suggested that etiolated tissues could be used for the proteomic analysis of thermo-responsiveness, in which PIF4/PIF7-independent regulators are also potentially involved.

### Quantitative Proteomic Analysis of Thermo-Responsiveness in *Arabidopsis*

**Figure 2:**
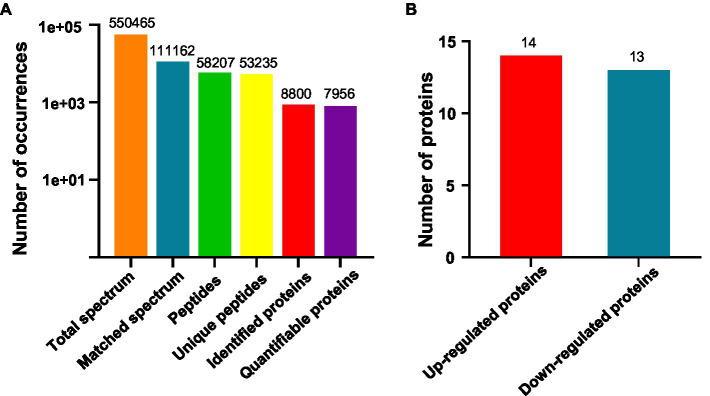
Quantitative proteomic study on thermo-responsiveness in *Arabidopsis*. Two-day-old *Arabidopsis* wild-type plants were treated at 22°C or 29°C for 3h and etiolated tissues were sampled for TMT (Tandem Mass Tags)-based quantitative proteomic analysis. Basic information on proteomics was shown in **(A)** and the number of differentially expressed proteins was shown in **(B)**.

We are interested in early thermo-responsive regulators in plants. Therefore, we treated 2-day-old etiolated WT plants grown at 22°C with warm temperature (29°C) for 3h in the dark and collected them for proteomic analysis. Firstly, we performed TMT-based quantitative proteomic analysis (Supplementary Figure S2). Totally 550,465 spectrums were obtained, among them, 111,162 spectrums matched to 58,207 peptides, representing 8,800 proteins, of which 7,956 proteins were quantifiable (Figure 2A). Among all the quantifiable proteins, there were only 14 proteins that were upregulated (Fold change >1.3, *p*<0.05) and 13 proteins that were downregulated (Fold change <1/1.3, *p*<0.05) by warm temperature treatments (Figure 2B and Supplementary Table S2), with their potential functions in thermo-responsiveness discussed later in the paper. Therefore, a short period of warm temperature treatment did not affect the accumulation level of most of the proteins in *Arabidopsis* proteomes, which is favorable for later phosphoproteomic studies.

### Phosphoproteomic Analysis of Thermo-Responsiveness in *Arabidopsis*

**Figure 3:**
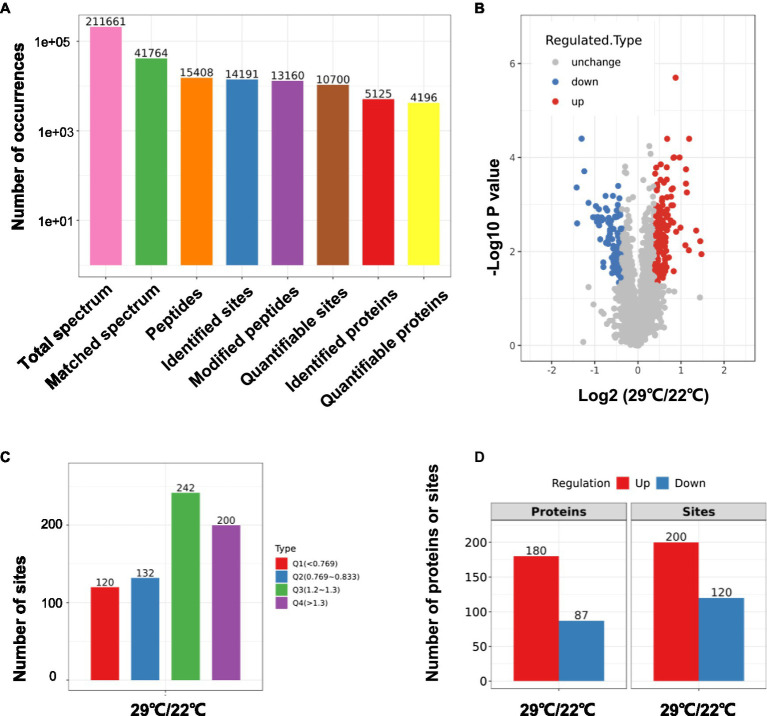
Phosphoproteomic study on thermo-responsiveness in *Arabidopsis*. Two-day-old *Arabidopsis* wild-type plants were treated at 22°C or 29°C for 3h, and etiolated tissues were sampled for TMT-based phosphoproteomic analysis. Basic information on proteomics was shown in **(A–C)** and the number of differentially phosphorylated proteins/sites (Q1 and Q4 in C) was shown in **(D)**.

**Figure 4:**
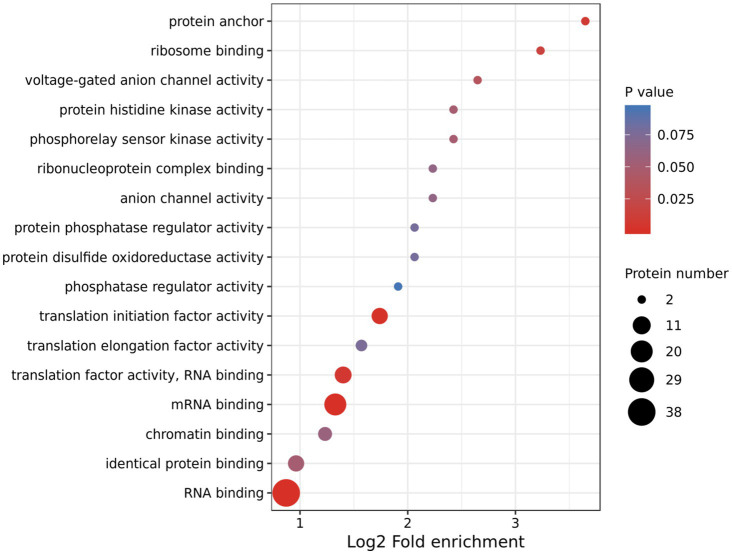
Gene Ontology (GO) analysis of differentially phosphorylated proteins by warm temperature. GO annotation was derived from the UniProt-GOA database or with the InterProScan software. The GO category Molecular Function is shown.

Using TMT-labeling coupled with phosphor-peptide enrichment and LC-MS/MS analysis, we compared phosphorylation modifications in etiolated seedlings between non-treated (22°C) and treated (29°C) samples (3h) in the dark. Totally 211,661 spectrums were obtained, among them, 41,764 spectrums matched to 15,408 peptides, representing 5,125 proteins, of which 4,196 proteins were quantifiable (Figure 3A). There were 13,160 modified peptides at 14,191 sites with 10,700 quantifiable sites (Figure 3A). Principle component analysis (PCA) showed a good reproducibility among three replicates of two comparisons (Supplementary Figure S3). Warm temperatures had a profound effect on protein phosphorylation in the tested samples (Figures 3B,C). There were 180 proteins that were upregulated (fold change >1.3, *p*<0.05), and 87 proteins that were downregulated (fold change <1/1.3, *p*<0.05) at phosphorylation level by warm temperature treatments at 200 and 120 sites, respectively (Figure 3D and Supplementary Table S3). GO (Gene Ontology) analysis of these 265 differentially phosphorylated proteins showed that phosphorelay sensor kinase activity, protein histidine kinase activity, protein phosphatase regulator activity, etc., were enriched (Figure 4 and Supplementary Figures S4, S5). These results revealed new potential regulators in the signaling pathway for thermo-responsiveness in plants.

### Validation the Phosphorylation Sites of ATL6

**Figure 5:**
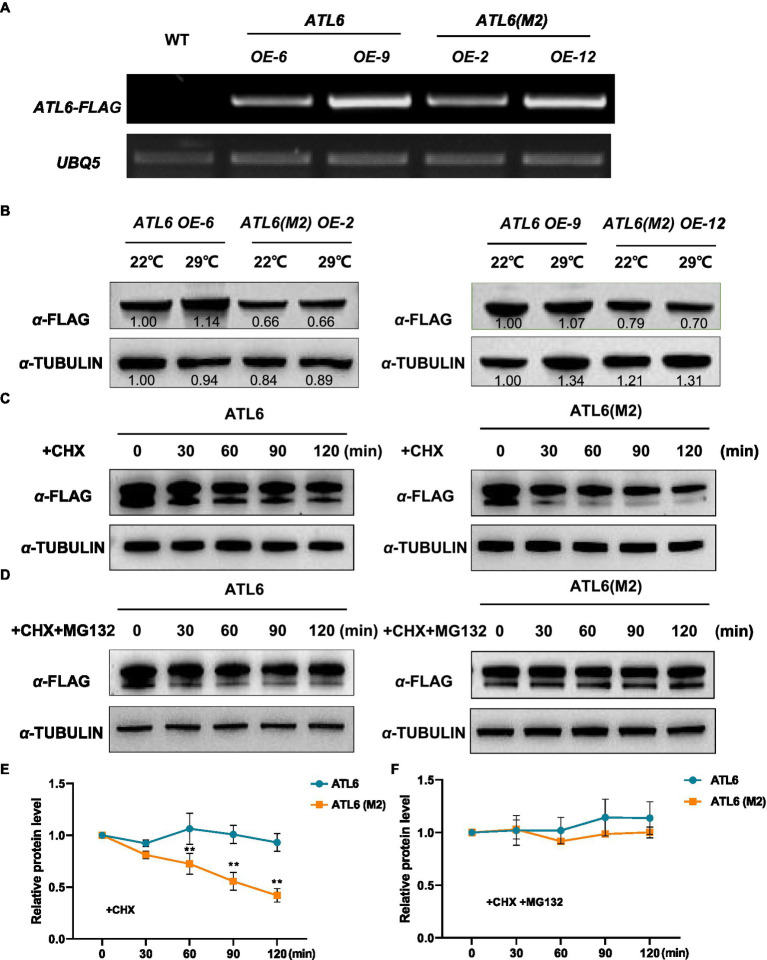
Protein stability of the native and mutated forms of ATL6 in response to warm temperature. The FLAG-tagged native form (*ATL6*) or the mutated form *ATL6(M2)* (S343A S357A) was overexpressed in *Arabidopsis*, and the transgene expression was validated by RT-PCR **(A)**. Two-day-old plants were grown at 22°C and 29°C for 3h, and the accumulation of ATL6-FLAG or ATL6(M2)-FLAG was checked by western blotting analysis **(B)**. The protein stability of the native or mutated form of ATL6 was investigated in the cell-free degradation assays **(C–F)**. Signal intensity of each band was quantified and normalized to that of the first sample. Relative protein level is the quantified signals of ATL6 normalized to that of TUBULIN from three western blots. The bars depict the *SE* (*n*=3). ^**^Significant in *t*-test (*p*<0.01). Cycloheximide (CHX) was used to inhibit protein synthesis and MG132 is an inhibitor of the 26S proteasome.

To validate the phosphoproteomic data, we focused on one differentially phosphorylated protein ATL6 (ARABIDOPSIS TOXICOS EN LEVADURA 6), which had two identified phosphorylation sites at 343 and 357, respectively (Supplementary Figure S6). Phosphorylation of ATL6 at S343 was upregulated by warm temperatures (fold change=1.6, *p*<0.05), while phosphorylation of ATL6 at S357 was not much affected (fold change=1.1, *p*>0.05). We overexpressed the FLAG-tagged native ATL6 in *Arabidopsis* and the IP-MS/MS results confirmed the occurrence of phosphorylations at these two sites (Supplementary Figure S7). We also overexpressed the mutated form ATL6(M2)-FLAG (S343A S357A) in *Arabidopsis*. Two transgenic lines were selected for each overexpression, with a comparable transgenic expression in between *ATL6-FLAG* and *ATL6(M2)-FLAG* overexpression plants for each comparison (Figure 5A). Western blotting analysis showed that comparing to the accumulation of ATL6-FLAG,the phosphorylation site mutations obviously reduced its accumulation both at 22°C and 29°C (Figure 5B). Subsequently, we performed cell-free degradation assays. The mutated form ATL6(M2)-FLAG degraded much faster than the native form ATL6-FLAG, which was inhibited by adding the 26S proteasome inhibitor MG132 (Figures 5C–F). We also checked the hypocotyl phenotypes of these transgenic plants in the dark. Overexpression of *ATL6-FLAG* slightly increased hypocotyl growth at both 22°C and 29°C while overexpression of *ATL6(M2)-FLAG* did not (Supplementary Figures S8A,B). These results supported that ATL6 is phosphorylated in *Arabidopsis* seedlings and phosphorylation of ATL6 increases its protein stability.

## Discussion

Previous results have shown that the heat stress response regulator heat shock factor 1 (HSF1) is also involved in controlling downstream gene expression during thermomorphogeneis (Cortijo et al., 2017). Multiprotein Bridging Factor 1c (MBF1c) is a transcriptional activator that is important for thermotolerance in *Arabidopsis*, and overexpression of *MBF1c* enhances the tolerance of transgenic plants to bacterial infection, heat, and osmotic stress (Suzuki et al., 2005, 2008, 2011). The MBF1c protein level is highly upregulated by warm temperature in the current study, which is consistent with its function in thermo-responses. It is well-known that auxin biosynthesis and signaling are essential for thermomorphogenesis (Casal and Balasubramanian, 2019). Auxin maxima are established by the auxin efflux carrier PIN-FORMED1 (PIN1), and NON-PHOTOTROPIC HYPOCOTYL 3 (NPH3)-like protein MACCHI-BOU 4 (MAB4) regulate PIN endocytosis and polarization (Furutani et al., 2011, 2014). The MAB4 protein level is upregulated by warm temperature (Supplementary Table S2), suggesting that auxin polar transport is also important for thermomorphogenesis. The gaseous phytohormone ethylene suppresses hypocotyl growth during skotomorphogenesis (Dubois et al., 2018). In contrast, under light conditions, ethylene promotes hypocotyl growth at 22°C but represses it at 28°C (Kim et al., 2021). At warm temperatures, the ethylene-activated transcription factor ethylene insensitive 3 (EIN3) directly induces the transcription of *ARABIDOPSIS PP2C CLADE D7* (*APD7*) gene encoding a protein phosphatase that inactivates the plasma membrane (PM) H^+^-ATPase SAUR19 (Chang et al., 2013; Kim et al., 2021). Indeed, the accumulation level of APD7 is upregulated by warm temperature (Supplementary Table S2). Alternative polyadenylation (APA) is an RNA-processing mechanism that generates distinct 3' termini on mRNAs and other RNA polymerase II transcripts (Tian and Manley, 2017). Pcf11p-similar protein 4 (PCFS4), an *Arabidopsis* homolog of yeast polyadenylation factor Protein 1 of Cleavage Factor 1 (*Pcf11p*), is responsible for *FLOWERING TIME CONTROL LOCUS A* (*FCA*) alternative processing and promotes flowering (Xing et al., 2008). Interestingly, the PCFS4 protein level is also upregulated by warm temperature (Supplementary Table S2), how APA regulates thermo-responses in plants awaits further investigation. Alternative splicing (AS) is another type of gene expression regulation; it is closely linked to plant responses to environmental stimuli including ambient temperature (Lin and Zhu, 2021). U1 small nuclear ribonucleoprotein (RNU1) is the smallest subcomplex of the spliceosome, a molecular machine for precursor mRNA (pre-mRNA) splicing (Chen et al., 2020). U2 small nuclear ribonucleoprotein auxiliary factor (U2AF) is another pre-mRNA splicing factor (Domon et al., 1998). RNU1 and U2AF protein levels are both upregulated by warm temperature, suggesting that they are involved in thermo-responsiveness by controlling AS in plants. Altogether, our quantitative proteomic analysis confirms the function of several proteins in thermomorphogenesis and provides new candidate thermo-responsive regulators for further future studies.

Phosphorylation is one of the most important post-translational modifications of proteins and regulates cellular processes in many signaling transduction pathways (Zhu and Li, 2013). We took the advantages of both TMT-labeling technology and phosphopeptide enrichment with TiO_2_ and found that 265 proteins (320 sites) were differentially regulated at phosphorylation level by warm temperatures (29°C; Supplementary Table S3). Phosphorelay sensor kinase activity, protein histidine kinase activity, protein phosphatase regulator activity, and phosphatase regulator activity are enriched among the differentially phosphorylated proteins (Figure 4), suggesting that these identified proteins function in the early signaling pathway during thermo-responses. One of the best studied signaling cascades is the phosphorelay regulated by MITOGEN-ACTIVATED PROTEIN KINASEs (MAPKs) family proteins (Pan and De Smet, 2020). The MAPK signaling cascade includes a MAPK KINASE KINASE (MAP3K), a MAPK KINASE (MAP2K), and a MAPK, which receives signals from the upstream receptor and transduces the signals to downstream transcription factors or effectors (Xu and Zhang, 2015). In the current study, the phosphorylation level of two receptor-like protein kinases, three MAP3Ks, and at least nine protein kinases, including the CBL-interacting serine/threonine-protein kinase 16 (CIPK16) and calcium-dependent protein kinase 6/29 (CPK6/29), are upregulated by warm temperatures (Supplementary Table S3), which supports that protein phosphorylation modification is important for thermo-responses in plants. MITOGEN-ACTIVATED PROTEIN KINASE KINASE KINASE KINASEs (MAP4Ks) are also present in plants although their targets are unknown and not necessarily only MAP3Ks (Pan and De Smet, 2020). In our study, the phosphorylation level of one MAP4K (MAP4Ka2) at two sites is upregulated by warm temperatures (Supplementary Table S3). Recently, the phosphoproteome of *Arabidopsis* subjecting to warm temperature (27°C) under the light rather than in the dark was examined. Totally 212 differentially phosphorylated sites, which mapped to 180 functionally diverse proteins, were identified, leading to the discovery of a new thermomorphogenic regulator MITOGEN-ACTIVATED PROTEIN KINASE KINASE KINASE KINASE4 (MAP4K4)/TARGET OF TEMPERATURE3 (TOT3; Vu et al., 2018, 2021). MAP4K4/TOT3 belongs to the same family as MAP4Ka2 and is potentially required for BR-regulated hypocotyl growth in darkness under warm temperature conditions, possibly through regulating the BZR1 activity (Vu et al., 2021). The function of MAP4Ka2 is not reported and whether MAP4Ka2 is involved in thermomorphogenesis needs further investigation in the future. Taken together, our phosphoproteomic analysis is complementary to previous phosphoproteomic studies and provides strong candidates for future studies on thermo-responses in plants.

Protein phosphorylation controls the protein activity, stability, turnover, subcellular localization, and interaction with partner proteins. Little is known about the phosphorylation events that govern thermomorphogenesis in plants. Even for the newly discovered MAP4K4/TOT3, it is still not known how this protein conveys warm temperature signals to brassinosteroid-mediated growth control (Vu et al., 2021). In the current study, as an example, we found that the phosphorylation of ATL6 may contribute to its protein stability since mutations in the identified phosphorylation sites conferred reduced stability of ATL6 both *in vivo* and *in vitro* (Figure 5). ATL6 encodes a plant-specific RING-type ubiquitin ligase that plays a critical role in carbon (C) and nitrogen (N) utilization (Sato et al., 2009; Maekawa et al., 2012). Recently, a receptor kinase FERONIA (FER) was found to be an essential component that modulates the phosphorylation status of ATL6, which may regulate the stability of 14-3-3 proteins in response to altered C/N ratios (Xu et al., 2019). It is interesting to know whether FER is also involved in thermomorphogenesis by regulating the phosphorylation of ATL6 at the identified sites in our study. The function of ATL6 in thermomorphogenesis is not yet known. Overexpression of *ATL6* slightly promotes hypocotyl growth (Supplementary Figure S8); further genetic experiments are needed to understand the function of ATL6 and its homologs in thermo-responsiveness. Since ATL6 is an E3 ubiquitin ligase, the identification of its substrates in thermomorphogenesis will be the key to understand the underlying molecular mechanisms.

In conclusion, our current quantitative proteomics and phosphoproteomic study have revealed several new potential regulatory components in plant thermomorphogenesis, which would expand our knowledge on understanding thermomo-responsiveness in plants beyond the well-studied pathways.

## Data Availability Statement

The datasets presented in this study can be found in online repositories. The names of the repository/repositories and accession number(s) can be found at: http://www.proteomexchange.org/, PXD027095, PXD027094.

## Author Contributions

YJS and JXL designed the experiments, analyzed the data, and wrote the paper. YJS, QYZ, and ZWY performed the experiments. All authors contributed to the article and approved the submitted version.

## Funding

This project was financially supported by grants from the National Natural Science Foundation of China (31872653), the Zhejiang Provincial Talent Program (2019R52005), and the Fundamental Research Funds for the Zhejiang Provincial Universities (2021XZZX023).

## Conflict of Interest

The authors declare that the research was conducted in the absence of any commercial or financial relationships that could be construed as a potential conflict of interest.

## Publisher’s Note

All claims expressed in this article are solely those of the authors and do not necessarily represent those of their affiliated organizations, or those of the publisher, the editors and the reviewers. Any product that may be evaluated in this article, or claim that may be made by its manufacturer, is not guaranteed or endorsed by the publisher.

## References

[ref1] Box M. S. Huang B. E. Domijan M. Jaeger K. E. Khattak A. K. Yoo S. J.. (2015). ELF3 controls thermoresponsive growth in *Arabidopsis*. Curr. Biol. 25, 194–199. doi: 10.1016/j.cub.2014.10.076, PMID: 25557663

[ref2] Casal J. J. Balasubramanian S. (2019). Thermomorphogenesis. Annu. Rev. Plant Biol. 70, 321–346. doi: 10.1146/annurev-arplant-050718-095919, PMID: 30786235

[ref3] Chang K. N. Zhong S. Weirauch M. T. Hon G. Pelizzola M. Li H.. (2013). Temporal transcriptional response to ethylene gas drives growth hormone cross-regulation in *Arabidopsis*. elife 2:e00675. doi: 10.7554/eLife.00675, PMID: 23795294 PMC3679525

[ref4] Chen M. X. Zhang K. L. Gao B. Yang J. F. Tian Y. Das D.. (2020). Phylogenetic comparison of 5' splice site determination in central spliceosomal proteins of the U1-70K gene family, in response to developmental cues and stress conditions. Plant J. 103, 357–378. doi: 10.1111/tpj.14735, PMID: 32133712

[ref5] Chen Z. Zhong W. Chen S. Zhou Y. Ji P. Gong Y.. (2021). TMT-based quantitative proteomics analyses of sterile/fertile anthers from a genic male-sterile line and its maintainer in cotton (*Gossypium hirsutum* L.). J. Proteome 10:232. doi: 10.1016/j.jprot.2020.104026, PMID: 33127528

[ref6] Chung B. Y. W. Balcerowicz M. Di Antonio M. Jaeger K. E. Geng F. Franaszek K.. (2020). An RNA thermoswitch regulates daytime growth in *Arabidopsis*. Nat. Plants 6, 522–532. doi: 10.1038/s41477-020-0633-3, PMID: 32284544 PMC7231574

[ref7] Clough S. J. Bent A. F. (1998). Floral dip: a simplified method for *Agrobacterium*-mediated transformation of *Arabidopsis thaliana*. Plant J. 16, 735–743. doi: 10.1046/j.1365-313x.1998.00343.x, PMID: 10069079

[ref8] Cortijo S. Charoensawan V. Brestovitsky A. Buning R. Ravarani C. Rhodes D.. (2017). Transcriptional regulation of the ambient temperature response by H2A.Z nucleosomes and HSF1 transcription factors in *Arabidopsis*. Mol. Plant 10, 1258–1273. doi: 10.1016/j.molp.2017.08.014, PMID: 28893714 PMC6175055

[ref9] Delker C. Sonntag L. James G. V. Janitza P. Ibanez C. Ziermann H.. (2014). The DET1-COP1-HY5 pathway constitutes a multipurpose signaling module regulating plant photomorphogenesis and thermomorphogenesis. Cell Rep. 9, 1983–1989. doi: 10.1016/j.celrep.2014.11.043, PMID: 25533339

[ref10] Ding L. Wang S. Song Z. T. Jiang Y. Han J. J. Lu S. J.. (2018). Two B-box domain proteins, BBX18 and BBX23, interact with ELF3 and regulate thermomorphogenesis in *Arabidopsis*. Cell Rep. 25, 1718–1728. doi: 10.1016/j.celrep.2018.10.060, PMID: 30428343

[ref11] Domon C. Lorkovic Z. J. Valcarcel J. Filipowicz W. (1998). Multiple forms of the U2 small nuclear ribonucleoprotein auxiliary factor U2AF subunits expressed in higher plants. J. Biol. Chem. 273, 34603–34610. doi: 10.1074/jbc.273.51.34603 9852132

[ref12] Dubois M. Van den Broeck L. Inze D. (2018). The pivotal role of ethylene in plant growth. Trends Plant Sci. 23, 311–323. doi: 10.1016/j.tplants.2018.01.003, PMID: 29428350 PMC5890734

[ref13] Fiorucci A. S. Galvao V. C. Ince Y. C. Boccaccini A. Goyal A. Allenbach Petrolati L.. (2020). PHYTOCHROME INTERACTING FACTOR 7 is important for early responses to elevated temperature in *Arabidopsis* seedlings. New Phytol. 226, 50–58. doi: 10.1111/nph.16316, PMID: 31705802 PMC7064998

[ref14] Franklin K. A. Lee S. H. Patel D. Kumar S. V. Spartz A. K. Gu C.. (2011). PHYTOCHROME-INTERACTING FACTOR 4 (PIF4) regulates auxin biosynthesis at high temperature. Proc. Natl. Acad. Sci. U. S. A. 108, 20231–20235. doi: 10.1073/pnas.1110682108, PMID: 22123947 PMC3250122

[ref15] Furutani M. Nakano Y. Tasaka M. (2014). MAB4-induced auxin sink generates local auxin gradients in *Arabidopsis* organ formation. Proc. Natl. Acad. Sci. U. S. A. 111, 1198–1203. doi: 10.1073/pnas.1316109111, PMID: 24395791 PMC3903239

[ref16] Furutani M. Sakamoto N. Yoshida S. Kajiwara T. Robert H. S. Friml J.. (2011). Polar-localized NPH3-like proteins regulate polarity and endocytosis of PIN-FORMED auxin efflux carriers. Development 138, 2069–2078. doi: 10.1242/dev.057745, PMID: 21490067

[ref17] Gangappa S. N. Kumar S. V. (2017). DET1 and HY5 control PIF4-mediated thermosensory elongation growth through distinct mechanisms. Cell Rep. 18, 344–351. doi: 10.1016/j.celrep.2016.12.046, PMID: 28076780 PMC5263232

[ref18] Ibanez C. Delker C. Martinez C. Buerstenbinder K. Janitza P. Lippmann R.. (2018). Brassinosteroids dominate hormonal regulation of plant thermomorphogenesis via BZR1. Curr. Biol. 28, 303–310. doi: 10.1016/j.cub.2017.11.077, PMID: 29337075

[ref19] Jung J. H. Barbosa A. D. Hutin S. Kumita J. R. Gao M. Derwort D.. (2020). A prion-like domain in ELF3 functions as a thermosensor in *Arabidopsis*. Nature 585, 256–260. doi: 10.1038/s41586-020-2644-7, PMID: 32848244

[ref20] Jung J. H. Domijan M. Klose C. Biswas S. Ezer D. Gao M.. (2016). Phytochromes function as thermosensors in *Arabidopsis*. Science 354, 886–889. doi: 10.1126/science.aaf6005, PMID: 27789797

[ref21] Kim J. Y. Park Y. J. Lee J. H. Kim Z. H. Park C. M. (2021). EIN3-mediated ethylene signaling attenuates auxin response during hypocotyl thermomorphogenesis. Plant Cell Physiol. pcab028. doi: 10.1093/pcp/pcab028, PMID: 33594435

[ref22] Koini M. A. Alvey L. Allen T. Tilley C. A. Harberd N. P. Whitelam G. C.. (2009). High temperature-medated adaptations in plant architecture require the bHLH transcription factor PIF4. Curr. Biol. 19, 408–413. doi: 10.1016/j.cub.2009.01.046, PMID: 19249207

[ref23] Kumar S. V. Lucyshyn D. Jaeger K. E. Alos E. Alvey E. Harberd N. P.. (2012). Transcription factor PIF4 controls the thermosensory activation of flowering. Nature 484, 242–245. doi: 10.1038/nature10928, PMID: 22437497 PMC4972390

[ref24] Legris M. Klose C. Burgie E. S. Rojas C. C. Neme M. Hiltbrunner A.. (2016). Phytochrome B integrates light and temperature signals in *Arabidopsis*. Science 354, 897–900. doi: 10.1126/science.aaf5656, PMID: 27789798

[ref25] Lin J. Y. Zhu Z. Q. (2021). Plant responses to high temperature: a view from pre-mRNA alternative splicing. Plant Mol. Biol. 105, 575–583. doi: 10.1007/s11103-021-01117-z, PMID: 33550520

[ref26] Maekawa S. Sato T. Asada Y. Yasuda S. Yoshida M. Chiba Y.. (2012). The *Arabidopsis* ubiquitin ligases ATL31 and ATL6 control the defense response as well as the carbon/nitrogen response. Plant Mol. Biol. 79, 217–227. doi: 10.1007/s11103-012-9907-0, PMID: 22481162

[ref27] Martinez C. Espinosa-Ruiz A. de Lucas M. Bernardo-Garcia S. Franco-Zorrilla J. M. Prat S. (2018). PIF4-induced BR synthesis is critical to diurnal and thermomorphogenic growth. EMBO J. 37:e99552. doi: 10.15252/embj.201899552, PMID: 30389669 PMC6276883

[ref28] Nieto C. Lopez-Salmeron V. Daviere J.-M. Prat S. (2015). ELF3-PIF4 interaction regulates plant growth independently of the evening complex. Curr. Biol. 25, 187–193. doi: 10.1016/j.cub.2014.10.070, PMID: 25557667

[ref29] Nomoto Y. Kubozono S. Yamashino T. Nakamichi N. Mizuno T. (2012). Circadian clock- and PIF4-controlled plant growth: a coincidence mechanism directly integrates a hormone signaling network into the photoperiodic control of plant architectures in *Arabidopsis thaliana*. Plant Cell Physiol. 53, 1950–1964. doi: 10.1093/pcp/pcs137, PMID: 23037003

[ref30] Oh E. Zhu J. Y. Wang Z. Y. (2012). Interaction between BZR1 and PIF4 integrates brassinosteroid and environmental responses. Nat. Cell Biol. 14, 802–809. doi: 10.1038/ncb2545, PMID: 22820378 PMC3703456

[ref31] Osterlund M. T. Hardtke C. S. Wei N. Deng X. W. (2000). Targeted destabilization of HY5 during light-regulated development of *Arabidopsis*. Nature 405, 462–466. doi: 10.1038/35013076, PMID: 10839542

[ref32] Pan L. X. De Smet I. (2020). Expanding the mitogen-activated protein kinase (MAPK) universe: an update on MAP4Ks. Front. Plant Sci. 11:1220. doi: 10.3389/fpls.2020.01220, PMID: 32849755 PMC7427426

[ref33] Park Y. J. Lee H. J. Ha J. H. Kim J. Y. Park C. M. (2017). COP1 conveys warm temperature information to hypocotyl thermomorphogenesis. New Phytol. 215, 269–280. doi: 10.1111/nph.14581, PMID: 28418582

[ref34] Paulo J. A. Schweppe D. K. (2021). Advances in quantitative high-throughput phosphoproteomics with sample multiplexing. Proteomics 21:16. doi: 10.1002/pmic.202000140, PMID: 33455035 PMC8209658

[ref35] Pham V. N. Kathare P. K. Huq E. (2018). Phytochromes and phytochrome interacting factors. Plant Physiol. 176, 1025–1038. doi: 10.1104/pp.17.01384, PMID: 29138351 PMC5813575

[ref36] Proveniers M. C. G. van Zanten M. (2013). High temperature acclimation through PIF4 signaling. Trends Plant Sci. 18, 59–64. doi: 10.1016/j.tplants.2012.09.002, PMID: 23040086

[ref37] Raschke A. Ibanez C. Ullrich K. K. Anwer M. U. Becker S. Gloeckner A.. (2015). Natural variants of ELF3 affect thermomorphogenesis by transcriptionally modulating PIF4-dependent auxin response genes. BMC Plant Biol. 15:197. doi: 10.1186/s12870-015-0566-6, PMID: 26269119 PMC4535396

[ref38] Sato T. Maekawa S. Yasuda S. Sonoda Y. Katoh E. Ichikawa T.. (2009). CNI1/ATL31, a RING-type ubiquitin ligase that functions in the carbon/nitrogen response for growth phase transition in *Arabidopsis* seedlings. Plant J. 60, 852–864. doi: 10.1111/j.1365-313X.2009.04006.x, PMID: 19702666

[ref39] Sun J. Qi L. Li Y. Chu J. Li C. (2012). PIF4-mediated activation of *YUCCA8* expression integrates temperature into the auxin pathway in regulating *Arabidopsis* hypocotyl growth. PLoS Genet. 8:e1002594. doi: 10.1371/journal.pgen.1002594, PMID: 22479194 PMC3315464

[ref40] Suzuki N. Bajad S. Shuman J. Shulaev V. Mittler R. (2008). The transcriptional co-activator MBF1c is a key regulator of thermotolerance in *Arabidopsis thaliana*. J. Biol. Chem. 283, 9269–9275. doi: 10.1074/jbc.M709187200, PMID: 18201973

[ref41] Suzuki N. Rizhsky L. Liang H. J. Shuman J. Shulaev V. Mittler R. (2005). Enhanced tolerance to environmental stress in transgenic plants expressing the transcriptional coactivator multiprotein bridging factor 1c. Plant Physiol. 139, 1313–1322. doi: 10.1104/pp.105.070110, PMID: 16244138 PMC1283768

[ref42] Suzuki N. Sejima H. Tam R. Schlauch K. Mittler R. (2011). Identification of the MBF1 heat-response regulon of *Arabidopsis thaliana*. Plant J. 66, 844–851. doi: 10.1111/j.1365-313X.2011.04550.x, PMID: 21457365 PMC4372994

[ref43] Thines B. Harmon F. G. (2010). Ambient temperature response establishes ELF3 as a required component of the core *Arabidopsis* circadian clock. Proc. Natl. Acad. Sci. U. S. A. 107, 3257–3262. doi: 10.1073/pnas.0911006107, PMID: 20133619 PMC2840299

[ref44] Tian B. Manley J. L. (2017). Alternative polyadenylation of mRNA precursors. Nat. Rev. Mol. Cell Biol. 18, 18–30. doi: 10.1038/nrm.2016.116, PMID: 27677860 PMC5483950

[ref45] Vu L. D. Gevaert K. De Smet I. (2019a). Feeling the heat: searching for plant thermosensors. Trends Plant Sci. 24, 210–219. doi: 10.1016/j.tplants.2018.11.004, PMID: 30573309

[ref46] Vu L. D. Xu X. Gevaert K. Smet I. (2019b). Developmental plasticity at high temperature. Plant Physiol. 181, 399–411. doi: 10.1104/pp.19.00652, PMID: 31363006 PMC6776856

[ref47] Vu L. D. Xu X. Zhu T. Pan L. van Zanten M. de Jong D.. (2021). The membrane-localized protein kinase MAP4K4/TOT3 regulates thermomorphogenesis. Nat. Commun. 12:2842. doi: 10.1038/s41467-021-23112-0, PMID: 33990595 PMC8121802

[ref48] Vu L. D. Zhu T. Verstraeten I. van de Cotte B. Sequencing I. W. G. Gevaert K.. (2018). Temperature-induced changes in the wheat phosphoproteome reveal temperature-regulated interconversion of phosphoforms. J. Exp. Bot. 69, 4609–4624. doi: 10.1093/jxb/ery204, PMID: 29939309 PMC6117581

[ref49] Wang P. Xue L. Batelli G. Lee S. Hou Y. J. Van Oosten M. J.. (2013). Quantitative phosphoproteomics identifies SnRK2 protein kinase substrates and reveals the effectors of abscisic acid action. Proc. Natl. Acad. Sci. U. S. A. 110, 11205–11210. doi: 10.1073/pnas.1308974110, PMID: 23776212 PMC3703982

[ref50] Xing D. Zhao H. Xu R. Li Q. Q. (2008). *Arabidopsis* PCFS4, a homologue of yeast polyadenylation factor Pcf11p, regulates *FCA* alternative processing and promotes flowering time. Plant J. 54, 899–910. doi: 10.1111/j.1365-313X.2008.03455.x, PMID: 18298670

[ref51] Xu G. Chen W. Song L. Chen Q. Zhang H. Liao H.. (2019). FERONIA phosphorylates E3 ubiquitin ligase ATL6 to modulate the stability of 14-3-3 proteins in response to the carbon/nitrogen ratio. J. Exp. Bot. 70, 6375–6388. doi: 10.1093/jxb/erz378, PMID: 31433471 PMC6859809

[ref52] Xu J. Zhang S. (2015). Mitogen-activated protein kinase cascades in signaling plant growth and development. Trends Plant Sci. 20, 56–64. doi: 10.1016/j.tplants.2014.10.001, PMID: 25457109

[ref53] Yang P. Li Y. He C. Yan J. Zhang W. Li X.. (2020). Phenotype and TMT-based quantitative proteomics analysis of *Brassica napus* reveals new insight into chlorophyll synthesis and chloroplast structure. J. Proteome 214:103621. doi: 10.1016/j.jprot.2019.103621, PMID: 31863931

[ref54] Zhang L. L. Li W. Tian Y. Y. Davis S. J. Liu J. X. (2021a). The E3 ligase XBAT35 mediates thermoresponsive hypocotyl growth by targeting ELF3 for degradation in *Arabidopsis*. J. Integr. Plant Biol. 63, 1097–1103. doi: 10.1111/jipb.13107 33963671

[ref55] Zhang L. L. Luo A. Davis S. J. Liu J. X. (2021b). Timing to grow: roles of clock in thermomorphogenesis. Trends Plant Sci. doi: 10.1016/j.tplants.2021.07.020, PMID: 34404586

[ref56] Zhang L. L. Shao Y. J. Ding L. Wang M. J. Davis S. J. Liu J. X. (2021c). XBAT31 regulates thermoresponsive hypocotyl growth through mediating degradation of the thermosensor ELF3 in *Arabidopsis*. Sci. Adv. 7:eabf4427. doi: 10.1126/sciadv.abf4427, PMID: 33962946 PMC8104893

[ref57] Zhao Z. Liu J. Jia R. Bao S. Haixia Chen X. (2019). Physiological and TMT-based proteomic analysis of oat early seedlings in response to alkali stress. J. Proteome 193, 10–26. doi: 10.1016/j.jprot.2018.12.018, PMID: 30576833

[ref58] Zhu L. Li N. (2013). Quantitation, networking, and function of protein phosphorylation in plant cell. Front. Plant Sci. 3:302. doi: 10.3389/fpls.2012.00302, PMID: 23316209 PMC3539650

