# Supplementary material for: Phosphoproteomic Analysis of Thermomorphogenic Responses in Arabidopsis
Source: Front Plant Sci. 2021 Sep 16;12:753148. doi: 10.3389/fpls.2021.753148 (PMC8481946; doi:10.3389/fpls.2021.753148)
Supplement: Supplementary file 4 [file Data_Sheet_1.PDF]

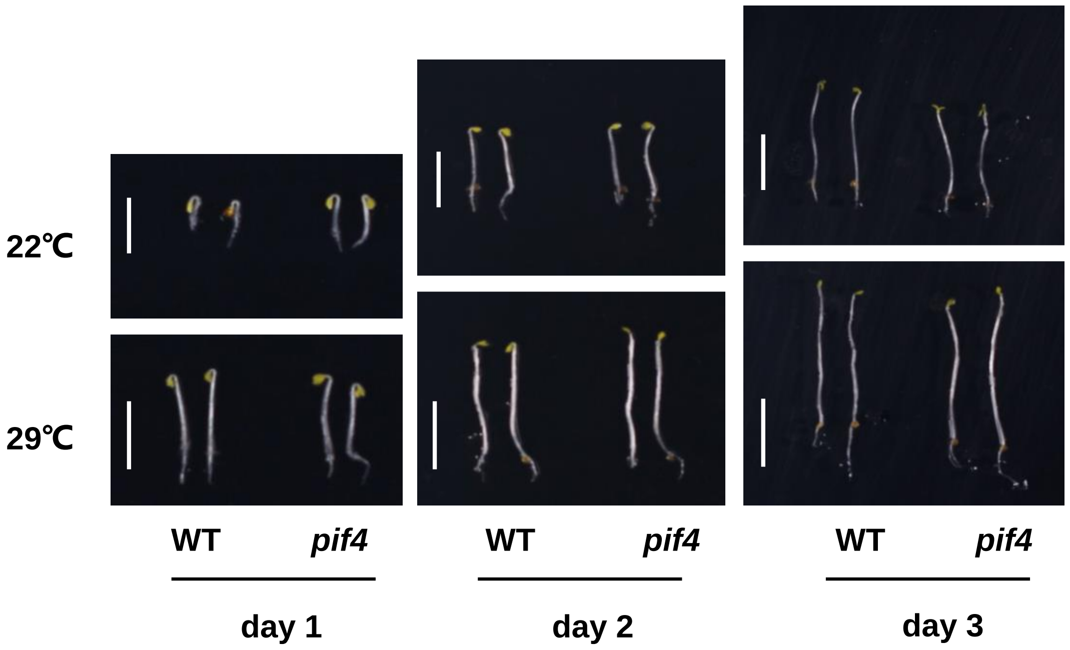

Figure S1. Thermoresponsive hypocotyl growth in the dark. Wild-type (WT) and *pif4* mutant (*pif4-101*) plants were grown at 22° C or 29° C in darkness for the indicated time and photographed. Bar = 5mm.

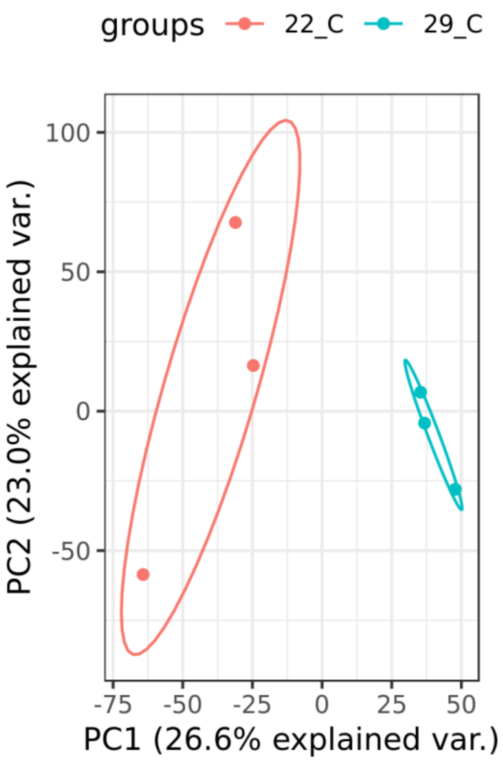

Figure S2. Principle Component Analysis (PCA) of samples for quantitative proteomics.

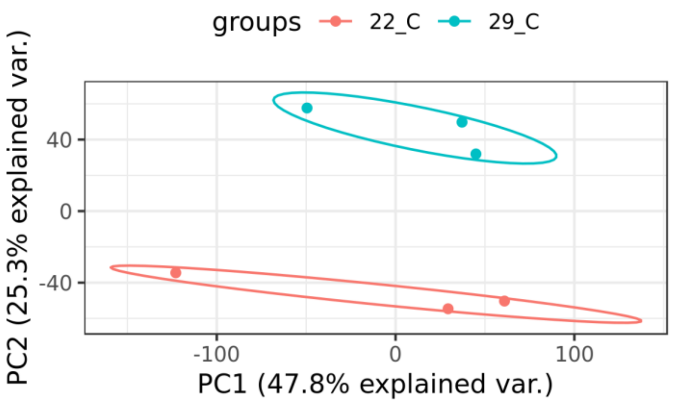

Figure S3. Principle Component Analysis (PCA) of samples for phosphoproteomics.

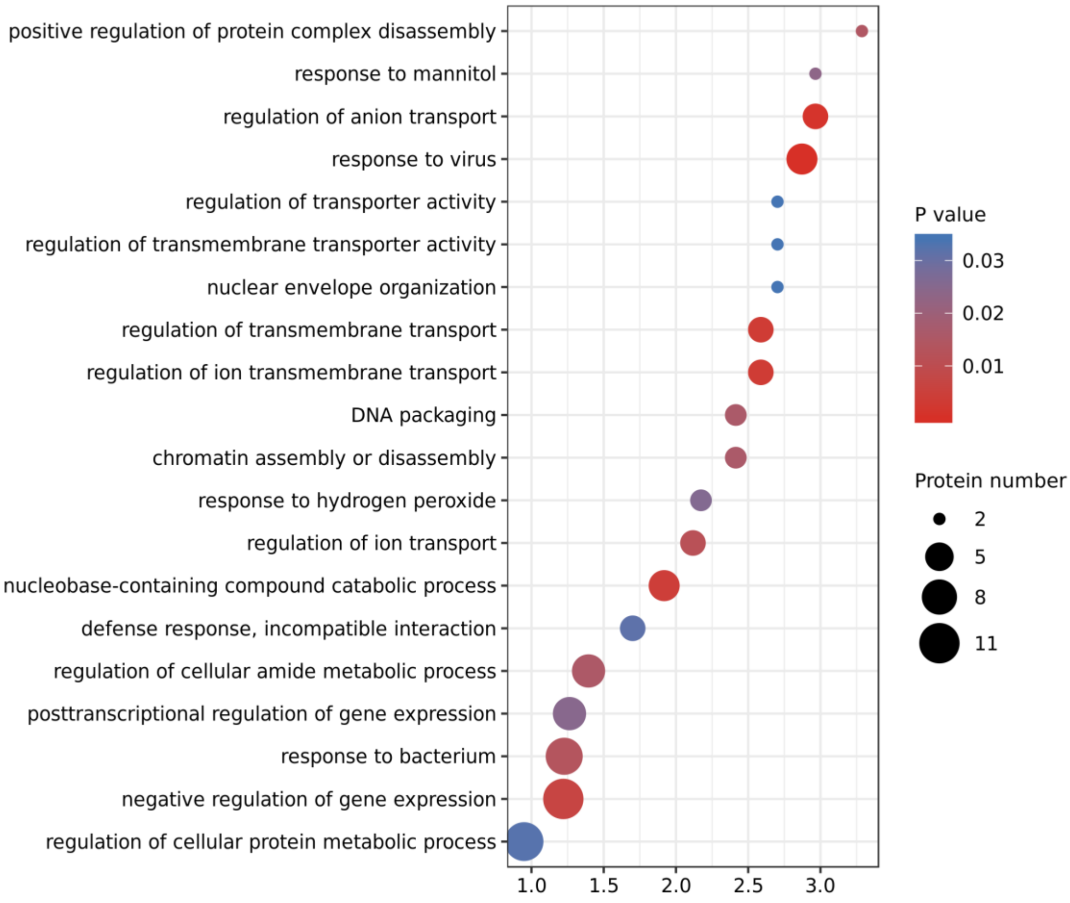

Figure S4. Gene Ontology (GO) analysis of differentially phosphorylated proteins by warm temperature (Biological Process).

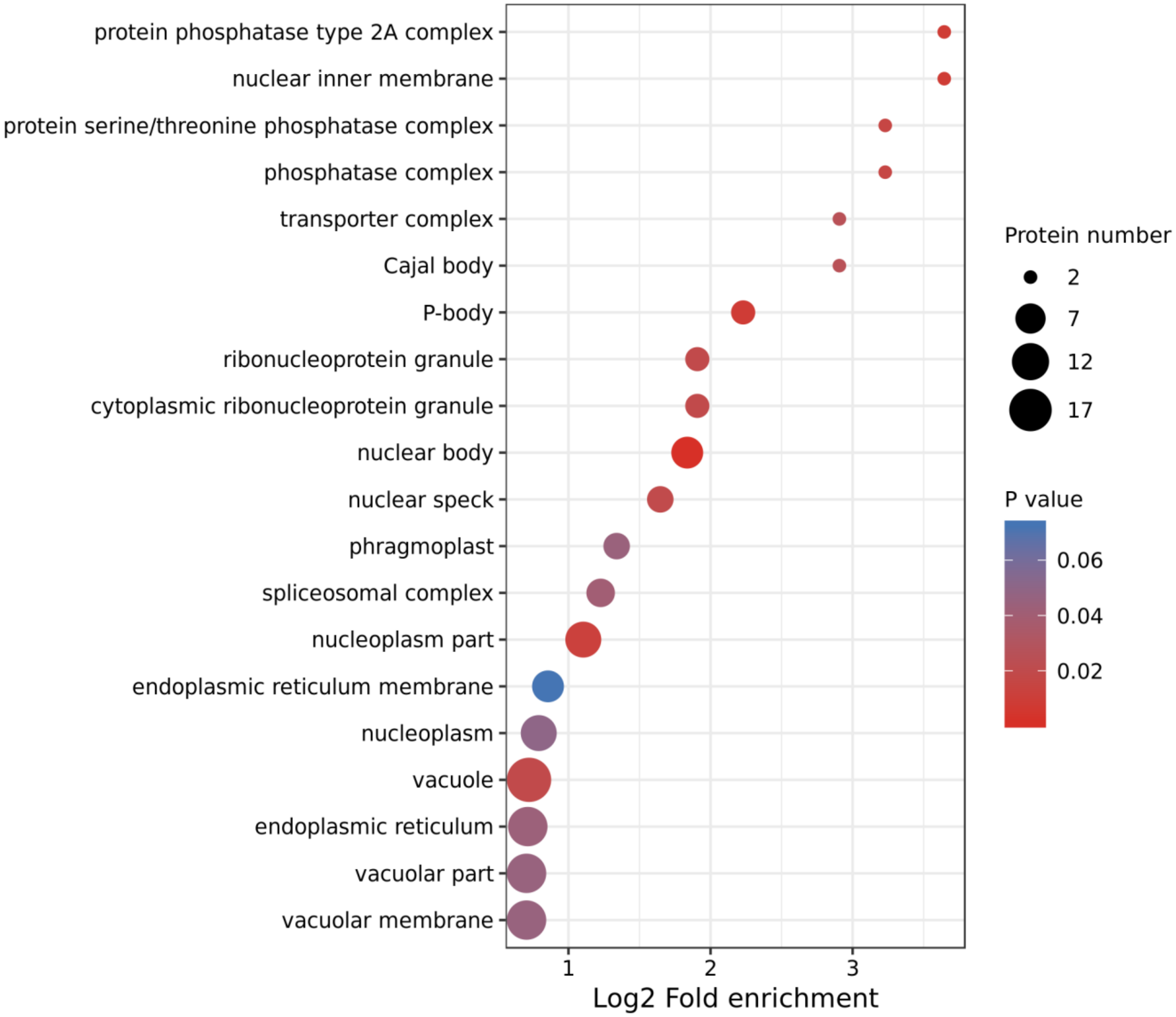

Figure S5. Gene Ontology (GO) analysis of differentially phosphorylated proteins by warm temperature (Cellular Component).

ATL6 S343 ASAVPNSTGS(0.02)DS(0.98)VR

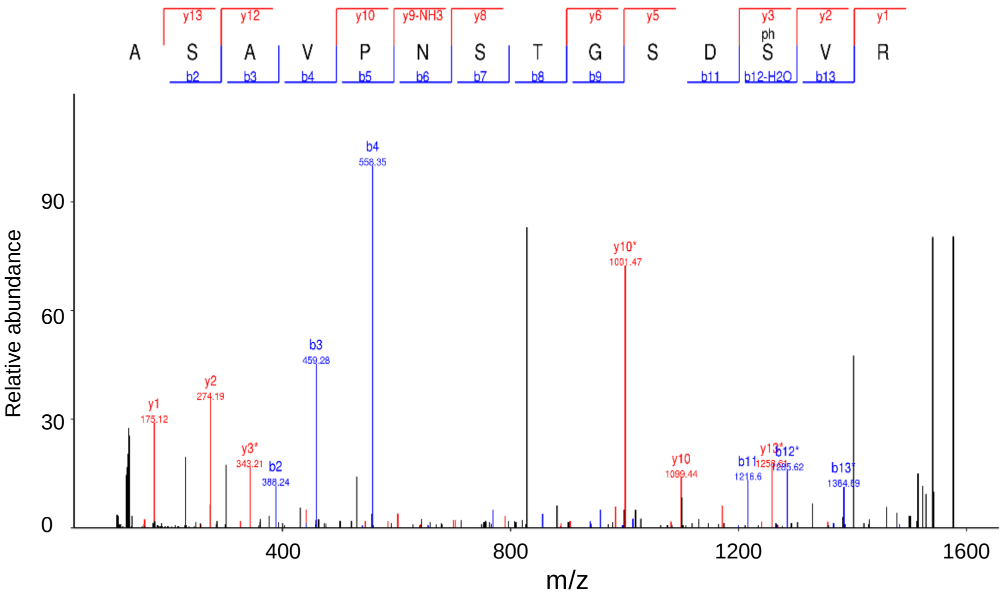

ATL6 S357 NAS(1)FLWR

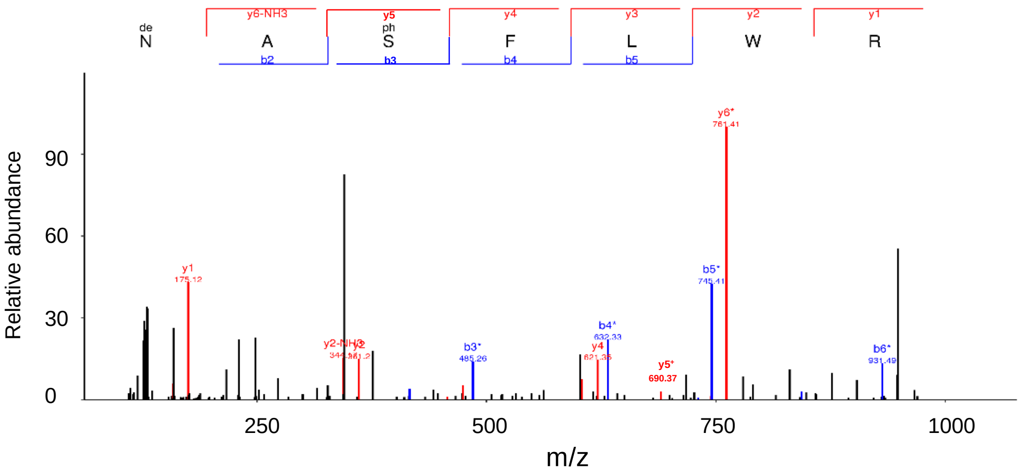

Figure S6. Mass spectrums for the phosphorylated peptides of ATL6 identified by LC-MS/MS.

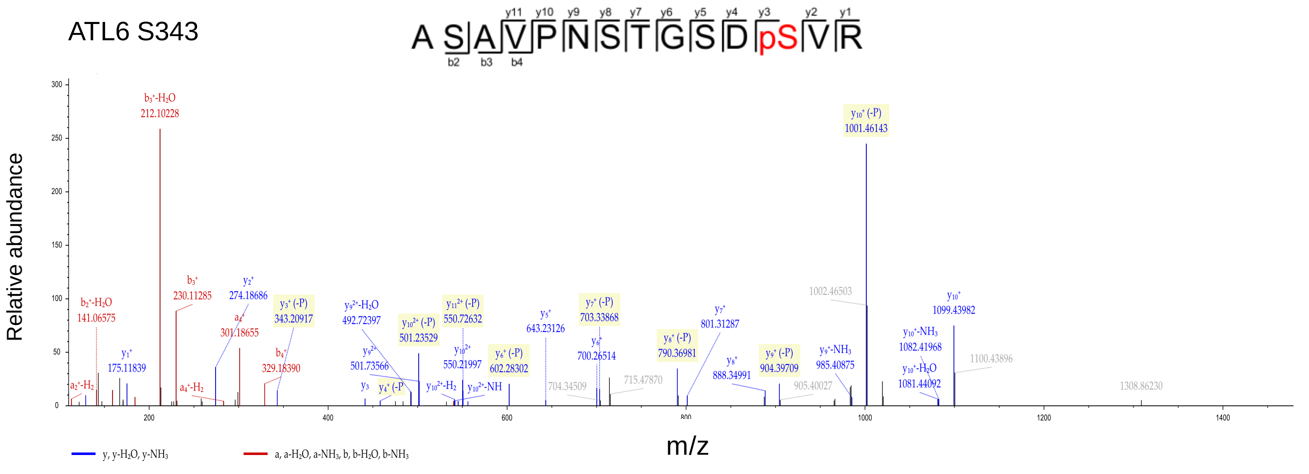

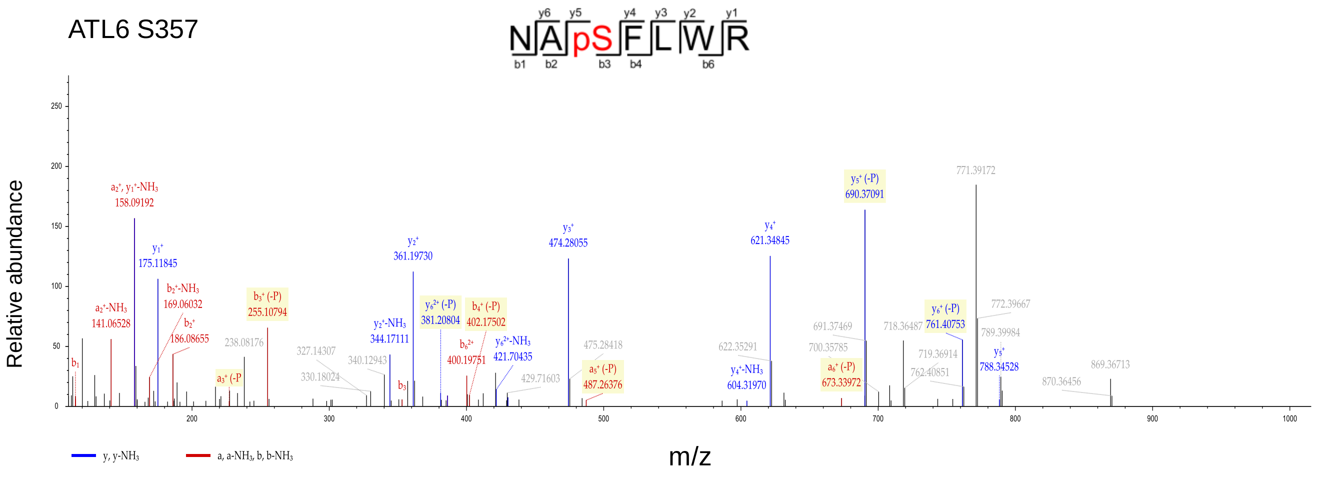

Figure S7. Mass Spectrums for the phosphorylated peptides of ATL6 identified by IP-MS/MS.

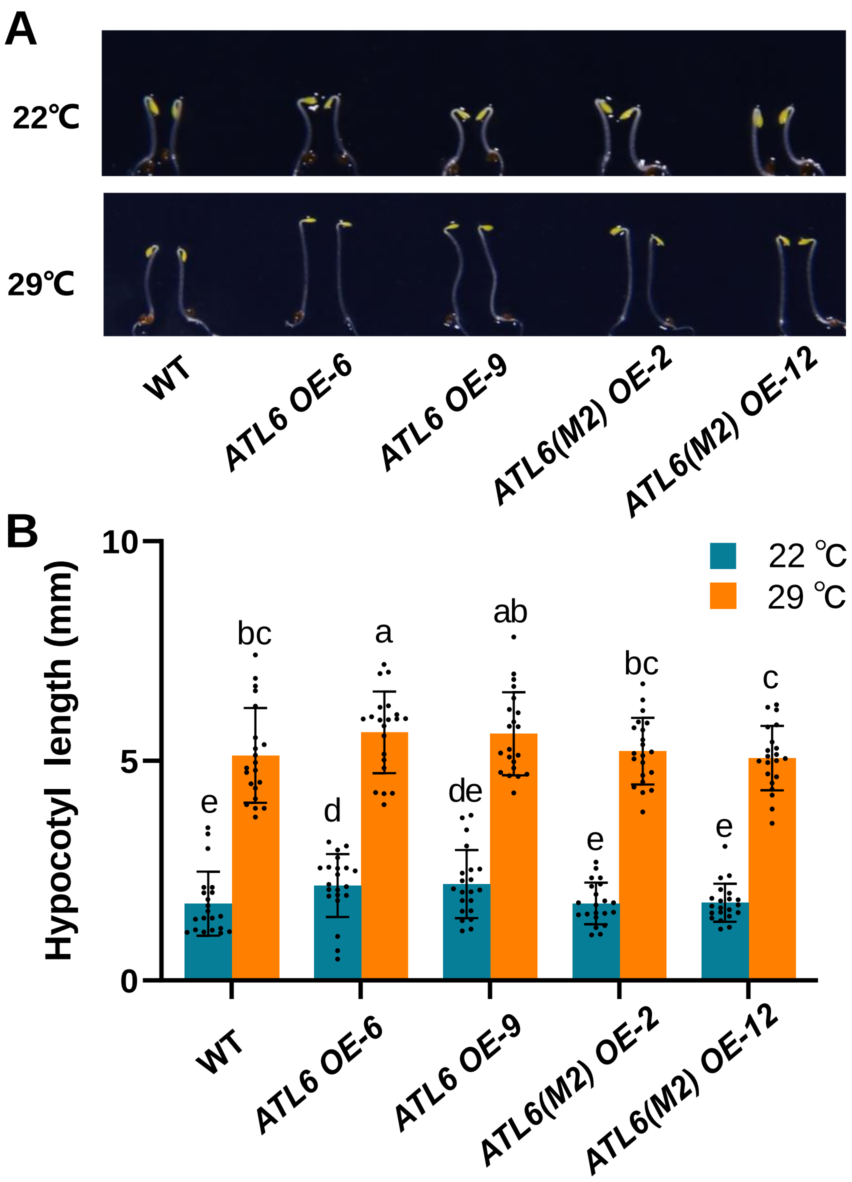

Figure S8. Thermo-responsive hypocotyl growth of *ATL6* overexpression plants in the dark. Wild-type (WT), ATL6-FLAG or ATL6(M2)-FLAG overexpression plants were grown at 22° C or 29° C in darkness for 2 days and photographed (A), and the hypocotyl length of each plant was subsequently measured (B). The bars depict the SD (n=18). Letters above the bars indicate significant differences as determined by HSD test ($P < 0.05$). Bar = 5mm. The native form ATL6-FLAG and the mutated form ATL6(M2)-FLAG (S343A S357A) were overexpressed in Arabidopsis wild-type (WT) background.
